# Diet after Stroke and Its Impact on the Components of Body Mass and Functional Fitness—A 4-Month Observation

**DOI:** 10.3390/nu11061227

**Published:** 2019-05-29

**Authors:** Justyna Leszczak, Ewelina Czenczek-Lewandowska, Grzegorz Przysada, Justyna Wyszyńska, Aneta Weres, Joanna Baran, Andrzej Kwolek, Artur Mazur

**Affiliations:** 1 Faculty of Medicine, University of Rzeszów, 35-959 Rzeszów, Poland; e.czenczek@univ.rzeszow.pl (E.C.-L.); g.przysada@interia.pl (G.P.); justyna.wyszynska@onet.pl (J.W.); budzinska2@tlen.pl (A.W.); joannabaran.ur@gmail.com (J.B.); kwoleka@o2.pl (A.K.); drmazur@poczta.onet.pl (A.M.); 2 Clinical Regional Hospital No. 2 in Rzeszów, Lwowska Street 60, 35-301 Rzeszów, Poland

**Keywords:** nutrition, diet, stroke, body composition, body mass index, functional fitness

## Abstract

The aim of the study was to assess the effect of various diets on BMI and selected components of body mass, i.e., fat mass (FAT%), visceral fat (VFAT level), muscle mass (PMM %), body water (TBW %), and functional fitness during a 4-month observation period. Examinations were conducted three times in a group of 100 people after a stroke. The study group was divided into four subgroups according to the type of diet applied. The components of body mass were assessed using the electrical bioimpedance method, and functional fitness using the Barthel scale, the Brunnström scale, and the modified Ashworth scale. Despite the fact that there were no significant differences among the diets applied, it was observed that each of them had a positive effect on the reduction of the mean BMI, FAT%, VFAT level, and the increase in TBW% and PMM%. At the same time, there was a significant improvement in the functional fitness of the hand and upper limb. Weight control and a change in eating habits after a stroke incident is extremely important as it promotes faster recovery and improved functional fitness.

## 1. Introduction

It is assumed that as many as 9 out of 10 strokes are associated with modifiable risk factors that can be influenced by appropriate therapy or lifestyle change [1]. Previous research confirms that compliance with a proper diet, in which the Mediterranean-style diet is particularly recommended, significantly reduces the risk of ischemic stroke and myocardial infarction [2,3]. Additionally, it has been confirmed that adherence to a DASH-style diet (described as reducing blood pressure) was associated with a lower incidence of stroke a middle-aged woman during 24 years of follow-up. However, it has been established that the Western dietary pattern was associated with increased stroke risk [4]. Recommendations place special emphasis on the consumption of large amounts of fruits and vegetables, reduced amounts of saturated fat, salt and sugar, and the complete elimination of alcohol and smoking. Fruits and vegetables are rich in antioxidants, which reduce damage to blood vessels, and potassium, which supports the control of hypertension. The fiber contained in vegetables promotes the lowering of cholesterol, and folic acid from leafy green vegetables and cereal products reduces the risk of stroke by up to 19% [5]. According to Deng et al., however, an increased risk is related to high consumption of red or highly processed meat [6]. The optimal supplement to a proper diet is an appropriately high level of physical activity, which promotes optimal control, maintains proper body weight, and reduces stress [7,8]. Regular nutritional education of patients after a stroke is extremely important, because, as described by numerous studies, most stroke patients have increased calorie intake and poor eating habits that they are reluctant to change [9].

It appears that healthy nutrition plays an important role in the prevention of stroke, but it also works in a favorable way after a vascular incident, because it can significantly facilitate and speed up the recovery process [10]. The consequences of stroke are numerous functional limitations, including: Reduction of muscle strength and tone, reduction of circulation in the upper and lower limbs, reduction of physical capacity and functional fitness, and often dysphagia [11]. Previous literature describes how excessive consumption of calories, reduced protein intake, and deficiencies of B and D vitamins and omega 3 fatty acids in people who have suffered a stroke may exacerbate existing disorders in the areas of physical, as well as metabolic and mental functioning [12]. The use of a properly balanced diet after a stroke can accelerate the effects of rehabilitation and prevent further complications after a stroke [13].

The aim of the study was to assess the impact of various diets on BMI, selected components of body mass, i.e., fat mass (FAT%), visceral fat (VFAT level), muscle mass (PMM %), body water (TBW %), and functional fitness during a 4-month observation period.

## 2. Materials and Methods

The research was carried out in the Rehabilitation Clinic with a Sub-Department of Early Rehabilitation of the Neurological Clinical Hospital in the period from June 2015 to March 2017 in a group of people after a stroke. The examinations were carried out three times. Examination I was performed on the day of admission to the Clinic, examination II on the day of discharge from the Clinic after 5 weeks, and examination III was carried out after 3 months from discharge from the Clinic during a follow-up visit.

The following qualification criteria were determined for the study: Stroke, ability to stand independently on the body mass composition analyzer, no higher psychiatric disorders, conscious, voluntary patient consent for participation in the study, and declaration of compliance with the recommended diet both in the hospital and post-hospital periods.

Patients were excluded from the study group who did not agree to participate in the study, who did not maintain an independent posture during the examination on the body composition analyzer, and those who did not follow the diet in the post-hospital period. Patients were also excluded if they had dysphagia or whose location of ischemic focus was found in the cerebellum and brain stem associated with impaired balance and those who had metal or electronic implants, epilepsy or injuries in the lower limbs from the onset of the stroke to the day of the study. In women, pregnancy and menstruation were also contraindications.

### 2.1. Subjects

The first study qualified 151 people after a stroke, but during the 4-month follow-up, due to the incompleteness of the results for analysis, 100 people were finally qualified (25 subjects did not apply the recommended diet in the post-hospital period; 14 subjects did not report for the follow-up visit without stating any reasons; 7 subjects withdrew without stating any reasons; 2 subjects—onset of epilepsy; 2 subjects—menstruation; 1 subject hospitalized due to another type of stroke). In the study group in the Rehabilitation Clinic in the hospital period, four different diets were used, i.e., basic diet (14 people), easily digestible (51 people), with restriction of easily digestible carbohydrates (*n* = 18), easily digestible low-protein (*n* = 17). This type of hospital diet used was selected individually for each patient based on the results of laboratory tests and the patient’s interview with a dietitian. Each subject participated in comprehensive hospital rehabilitation, and after discharge in outpatient or community rehabilitation. On the day of discharge (test II), the patient received dietary recommendations and sample menus, which the patient was recommended to use in the post-hospital period. On the day of the follow-up visit (study III) within 3 months after leaving the hospital, the patient had to declare whether he/she had been following the recommended diet.

#### 2.1.1. Basic Diet (D1)

This diet consisted of using 4–5 meals a day with a total value of 2000 kcal (medium standards were adopted with low physical activity, 1800 kcal for women, and 2270 kcal for men). The protein was 15.5%, fat 9.5%, and carbohydrates 55% of the sum of daily energy requirement.

#### 2.1.2. Easily Digestible Diet (D2)

This diet consisted of applying 4–5 meals a day with a total value of 2200 to 2500 kcal. The diet consisted in the selection of easily digestible dishes that were boiled or steamed. The diet reduced the supply of dietary fiber to 25 g, by choosing the right vegetables and fruits, making purées and juices, and the use of refined cereal products. The protein was 15.5%, fat 29.5% and carbohydrates 55% of the sum of the daily energy requirement.

#### 2.1.3. Diet with Restriction of Easily Digestible Carbohydrates (D3)

This diet consisted of applying 4–6 meals a day with a total value of 2000 to 2200 kcal. It was a diet with a low glycemic index and consisted of even distribution of carbohydrates across meals and the reduction or exclusion of carbohydrate-containing products. The diet was dedicated primarily to patients diagnosed with diabetes. Carbohydrates accounted for 40–50%, protein 15–20% and fat 30– 35% of the sum of the daily energy requirement. The diet consisted in limiting easily digestible carbohydrates, i.e., glucose, fructose, sucrose, and increasing the supply of complex carbohydrates, i.e., dietary fiber and starch.

#### 2.1.4. Easily Digestible Low-Protein Diet (D4)

This diet consisted of applying 4–6 meals a day with a total value of 2000 to 2200 kcal. It was based on the reduction of protein in the diet from 40 g to 20 g. The purpose of the diet was to reduce excessive production of protein metabolism products that are toxic to the body, protection of diseased organs—liver and kidneys—while maintaining a good nutritional status of the patient by providing the necessary amount of energy and nutrients. The protein was 8%, fat 30%, and carbohydrates 62% of the sum of the daily energy requirement.

### 2.2. Assessments

Selected body composition components, i.e., fat mass (FAT%), visceral fat (VFAT level), muscle mass (PMM %), body water (TBW %), and body mass index (BMI) were assessed with the Tanita MC 780 MA analyzer, using the method of electrical bio-impedance (BIA) measurements.

Body height was measured with an accuracy up to 0.1 cm using a PORTSTAND 210 portable stadiometer. The subjects, in underwear and with no shoes, were instructed to assume a straight body posture with eyes directed straight ahead. All of the examinations were performed by a single physiotherapist in the morning.

#### Functional Fitness Test

The Barthel index is used to assess everyday activities. It is divided into 10 studied areas of ability, i.e., eating, bathing, grooming, dressing, controlling the anal sphincter, controlling the bladder sphincter, using the toilet, moving (from bed to chair and back), and moving on a flat surface and on stairs. Skills are rated on a scale of 0 to 100 points, where a higher score indicates higher functional status of the patient [14,15].

The Brunnström scale is used to assess upper and lower limb movement. It is built from six points assessing the capabilities of paretic limbs. A higher grade indicates better movement capability of the limb under test [16,17].

The modified Ashworth scale is used to assess increased muscle tone during spasticity. It is a modified six-level scale from 0 to 4, including the 1+ tone level. A lower level of the scale indicates lower muscle tone in the examined limb [18].

### 2.3. Statistical Analysis

The analysis uses descriptive statistics (mean, standard deviation, standard error, minimum, maximum, 95% confidence interval for the mean).

In the study of the occurrence of differences between particular stages of the study (I, II, III), a one-way analysis of variance was used. The ANOVA parameter test was used.

The Kruskal–Wallis test was used because the distribution of the “functional fitness” variables was not a normal distribution (verified by the Kolmogorov–Smirnov and Shapiro–Wilk tests). The significance level was *p* < 0.05. The statistical power is 0.9, i.e., the maximum error is 10%. All calculations and statistical analyses were performed using the STATISTICA ver. 10.0 (StatSoft).

### 2.4. Ethics

The study was conducted in accordance with the ethical rules of the Helsinki Declaration. The study protocol was approved by the Local Bioethics Commission (Consent No. 2015/10/03) and by all appropriate administrative bodies. Each patient expressed voluntary written consent to participate in the study and was informed about the examination procedure.

## 3. Results

### Characteristics of the Study Group

In the study group, there were 100 people after a stroke who participated in three examinations (study I, II, III). In the study group, 4 types of diets were applied: Basic diet (D1), 14% (*n* = 14) of people, easily digestible diet (D2) 51% (*n* = 51) of people, diet with a restriction of easily digestible carbohydrates (D3) 18% (*n* = 18) of people, easily digestible low-protein diet (D4), 17% (*n* = 17) of people. Fifty-eight percent (*n* = 58) of the group were men, and 42% (*n* = 42) women, aged from 19 to 88 years old (58.34 ± 14.54). Ischemic stroke occurred in 82 people (82%) and hemorrhagic stroke in 18 people (18%). Descriptive characteristics of the study sample are shown below (Table 1).

There were no statistically significant differences between the diets applied: D1, D2, D3, D4, and body mass components. For this reason, data from the general group were subject to further analysis. The lack of differences may result from the fact that the diets applied were similar to each other in terms of caloric content. A decrease in BMI was observed, starting from examination I (Mean = 27.567), through examination II (Mean = 27.491) and examination III (Mean = 27.480). The value of BMI decreased mainly in the period between examinations I and II and was maintained until examination III. A similar relationship was noted in the case of other values, with a reduction from examination I to examination II followed by a modest uptick by examination III, e.g., FAT values, i.e., examination I (Mean = 26.421), examination II (Mean = 25.757), examination III (Mean = 25.960). The level of VFAT also decreased during the application of diets in the hospital period: Examination I (Mean = 10.02), examination II (Mean = 9.87), and the lowered level was partially maintained during the patients’ stay at home until examination III (Mean = 9.94). The range of PMM increased during the patients’ stay in the hospital clinic: Examination I (Mean = 69.88) until discharge in the course of examination II (Mean = 70.52). It was observed that the higher PMM value was partially maintained 3 months after discharge during examination III (Mean = 70.26). TBW increased compared to study I (Mean = 52.18), and there were no signs of dehydration of stroke patients in examination II (Mean = 52.58) and in examination III (Mean = 52.46). Despite the fact that the observed differences did not show significant statistical significance, a positive effect of the diets on the components of body mass was demonstrated. Detailed results are shown in Table 2.

A one-way analysis of variance showed that there are no statistically significant differences in the measurements of the components of body mass according to the individual stages of the examinations (I, II, III) (Table 3).

The greatest improvement in functional fitness of the upper limbs according to the Brunnström scale was demonstrated in people who were on an easily digestible low-protein diet (0.59 ± 0.51), and to a lesser extent in people on a diet with a restriction of easily digestible carbohydrates (0.39 ± 0.61) and an easily digestible diet (0.25 ± 0.44), and to the lowest extent in people on a basic diet (0.07 ± 0.27). Patients on an easily digestible low-protein diet achieved better results in terms of muscle tone of the hand and upper limb according to the Ashworth scale (0.67 ± 0.79) than those following other types of diet (0.53 ± 0.62) (Table 4).

## 4. Discussion

Stroke is the second leading cause of mortality in the world and the third leading cause of disability. It is increasingly affecting people below 65 years of age, who account for about 30% of all cases [19]. Many negative health consequences of stroke, including reduced functional and mental fitness as a result of the existing muscle paresis, may be controlled by a change in lifestyle, as part of which it is extremely important to improve eating habits [20]. The available literature describes the positive role of healthy nutrition in the prevention of cardiovascular diseases, while much less research focuses on the role of nutrition in recovery and functional fitness after a stroke incident. Although many studies describe the role of healthy diet in stroke, the presented study is one of the first that takes into account both the type of diet used in the hospital and environmental conditions as well as its connection with the convalescence of the patient.

So far, many risk factors have been recognized, both modifiable (obesity, poor eating habits, hypertension, low physical activity, smoking, drinking alcohol) and non-modifiable (age, sex, race, genetic determinants). Despite the widespread knowledge on this topic, many stroke survivors are not aware or do not care enough about maintaining a healthy lifestyle. Numerous studies show that most of them have problems with improving their health habits even after the incident. According to Redfern, who analyzed the health behaviors of stroke survivors during the year after their stroke, as many as 22% of them still smoked cigarettes, 36% were obese, and 4% still drank alcohol in excessive amounts [21]. In a study by Bailey et al., healthy adults significantly more often consumed 1 or more fruit and 1 or more vegetable daily and fulfilled the required norms of physical activity in comparison to people after a stroke [22]. Yuki, on the other hand, states that from 25% to 65% of patients after a stroke did not know any risk factor for this disorder [23].

From previous observations, it appears that an abnormal diet rich in high-fat products, as well as salt or sugar, plays an important role in the development of cardiovascular diseases. These dietary components foster further complications such as obesity, hypertension, and hyperlipidemia, which significantly accelerate calcification of the atherosclerotic plaque and increase the risk of stroke. Strazzullo et al., analyzing a cohort of 2.2 million people after a stroke, confirmed the close relationship between excessive body mass and the occurrence of stroke [24]. Despite controversial results suggesting an “obesity paradox” in stroke, which is indicative of reduced mortality of obese patients, obesity is a predisposed risk factor for stroke, and weight loss in overweight or obese patients is still recommended primarily for the prevention of primary stroke [25]. Control of weight and maintaining it in the proper range is equally important after a stroke incident, and the main regulatory factor is diet.

In the study, although no significant differences were found among the different types of diet, it should be emphasized that each of them had a beneficial effect on the reduction of BMI and individual components of body mass, which are not conducive to health, i.e., BMI, FAT%, VFAT level. At the same time, it was observed that after a 4-month period of compliance with dietary recommendations, patients increased their bodily hydration level (TBW%) and increased their muscle mass. These results favored better effects of rehabilitation, which was confirmed by a significant improvement in the functional fitness of the upper and lower limbs measured with the Brunnström and Ashworth scales. A relationship was observed between higher functional fitness of patients primarily with an easily digestible low-protein diet. The diet of people after a stroke should be subject to strict control and caloric planning, in particular in patients who have been found to have eating disabilities, i.e., malnutrition or overweight or obesity. As in our own study, Serra emphasizes that adhering to nutritional recommendations may benefit recovery and rehabilitation [26].

There are very few reports confirming the influence of BMI on the return to functional fitness in the period of convalescence after stroke. Kawase et al. found that underweight patients (BMI <18.5 kg/m2) had the lowest functional level in both the hospitalization and discharge periods assessed on the Rankin scale score, whereas the results of patients with normal weight and obesity did not differ significantly [27]. Similar conclusions are described by Sun et al. who reported a significantly lower improvement in the functional fitness of underweight patients [28]. As in our own study, the authors confirm the importance of constant weight control in patients after a stroke and the need to maintain it in the recommended range for age and gender.

Patients after a stroke have a number of changes in the individual components of body mass. Lazoura et al., among others, carried out a detailed analysis of these in the period of 1 year from stroke. Both men and women showed a significant decrease in mineral density in the total-body and paretic lower-limb areas, while in contrast with our own study, there was a significant increase in fat mass gain. Such changes may be associated with further complications, such as injuries due to falls and easier susceptibility to fractures [29]. From the point of view of the return to functional fitness, muscle tissue mass plays a key role. There is growing evidence that patients after a stroke are more susceptible to losing it, which is detrimental to the effects of rehabilitation and prolongs the period of hospitalization. Loss of muscle tissue is a complicated process explained primarily by feeding problems and motor dysfunction [30]. At the same time, it contributes to the loss of muscle strength, i.e., sarcopenia, which significantly reduces even the ability to move independently and increases the risk of falls [31].

Numerous functional problems of patients after a stroke can affect not only the musculoskeletal system but also the masticatory system, whose dysfunctions significantly impair the ability to chew, bite, and swallow solid foods. Dysphagia is described as a complication related to malnutrition and dehydration of the body, and musculoskeletal dysfunctions (including paresis of, for example, limbs, making it difficult to adopt the right position to eat food) [32,33] It is estimated that up to 50% of stroke patients may need outside help with eating and 10% even require complete outside help [34]. In the study, the study group excluded patients with dysphagia who need complete help with food intake; however, the problem of dysphagia is worth a separate and broader analysis. Rehabilitation of patients after a stroke should, in particular, take into account the ability to consume food, including maintaining the correct posture while eating, improving the gripping and manipulative functions of the hand and upper limb, as well as functional improvement of the masticatory organ [35]. These abilities, like the proper choice of diet, can directly affect the degree of nutrition and functioning of the patient [36].

## 5. Conclusions

The application of a healthy diet after a stroke should be obligatorily observed by every patient, as it has a beneficial effect on weight control and improvement of functional fitness. Parallel nutrition education and appropriately targeted rehabilitation is a guarantee of a faster recovery after a stroke.

## 6. Limitation of the Study

Future research should be conducted on a larger number of patients after a stroke and should be extended with long-term observation of eating habits beyond the first year after incidence of stroke. Additionally, the different genetic and environmental factors should be analyzed, i.e., the time from stroke, sex, age and type of stroke, because presumably they could affect the final results. A limitation of the presented study was the lack of opportunity to examine people before the incident as well as the lack of information about their previous eating habits.

## Figures and Tables

**Table 1:** Characteristics of the study group.

Variable	Values			
	Side of Paresis		Total	*p*
	Left	Rights		
	N (%)	N (%)	N (%)	
Diet ^a^				
Basic diet (D1)	7 (13.2)	7 (14.9)	14 (14)	0.7815
Easily digestible diet (D2)	25 (47.2)	26 (55.3)	51 (51)	
Diet with restriction of easily digestible carbohydrates (D3)	11 (20.8)	7 (14.9)	18 (18)	
Easily digestible low-protein diet (D4)	10 (18.9)	7 (14.9)	17 (17)	
**Sex ^a^**				
Female	18 (34.0)	24 (51.1)	42 (42)	0.0837
Male	35 (66.0)	23 (48.9)	58 (58)	
**Time from stroke ^a^**				
<6 months	19 (35.8)	9 (19.1)	28 (28)	0.1720
6–12 months	8 (15.1)	10 (21.3)	18 (18)	
>12 months	26 (49.1)	28 (59.6)	54 (54)	
**Type of stroke ^a^**				
Ischemic	40 (75.5)	42 (89.4)	82 (82)	0.0712
Hemorrhagic	13 (24.5)	5 (10.6)	18 (18)	
**Age (19–88 years) ^b^**	58.34 (14.54)	60.81 (14.46)	59.50 (14.48)	0.3975
**Age ^a^**				
19–50 years	13 (24.5)	10 (21.3)	23 (23)	0.5628
51–65 years	24 (45.3)	18 (38.3)	42 (42)	
66–88 years	16 (30.2)	19 (40.4)	35 (35)	

Data are expressed as: a—*n* (%); b—(SD); test chi2 for diet, sex, time from stroke, type of stroke; test t for age.

**Table 2:** Diets and body mass components in examinations I, II, and III.

	N	Mean	SD	SE	95% Cl	Min	Max	*p*
**BMI, and diets applied**								
Exam I	100	27.56	4.74	0.47	26.62–28.50	18.70	41.40	0.329
Exam II	100	27.49	4.64	0.46	26.56–28.41	18.60	41.40	0.238
Exam III	100	27.48	4.78	0.47	26.53–28.42	18.50	42.80	0.297
Total	300	27.51	4.70	0.27	26.97–28.04	18.50	42.80	0.990
**FAT [%], and diets applied**								
Exam I	100	26.42	7.34	0.73	24.96–27.87	13.80	43.50	0.250
Exam II	100	25.75	7.25	0.72	24.31–27.19	11.46	43.65	0.265
Exam III	100	25.95	7.39	0.73	24.49–27.42	11.46	42.43	0.346
Total	300	26.04	7.31	0.42	25.21–26.87	11.46	43.65	0.806
**VFAT [level], and diets applied**								
Exam I	100	10.02	4.590	0.459	9.11–10.93	1	21	0.193
Exam II	100	9.87	4.539	0.454	8.97–10.77	1	21	0.133
Exam III	100	9.92	4.614	0.461	9.00–10.84	1	21	0.146
Total	300	9.94	4.566	0.264	9.42–10.46	1	21	0.973
**TBW [%], and diets applied**								
Exam I	100	52.18	5.51	0.55	51.09–53.27	39.52	64.57	0.196
Exam II	100	52.58	5.44	0.54	51.50–53.66	39.95	64.50	0.196
Exam III	100	52.46	5.50	0.55	51.37–53.56	40.67	65.54	0.223
Total	300	52.41	5.47	0.31	51.79–53.03	39.52	65.54	0.867
**PMM [%], and diets applied**								
Exam I	100	69.88	6.98	0.69	68.49–71.26	53.58	81.88	0,.249
Exam II	100	70.52	6.90	0.69	69.15–71.89	53.51	84.14	0.271
Exam III	100	70.26	6.97	0.69	68.87–71.64	54.87	84.14	0.319
Total	300	70.22	6.93	0.40	69.43–71.01	53.51	84.14	0.804

BMI: Body mass index; FAT: Fat mass; VFAT: Visceral fat; TBW: Body water; PMM: Muscle mass; N: Number of subjects; SD: Standard deviation; SE: Standard error; CI: Confidence interval; Min: Minimum; Max: Maximum value.

**Table 3:** Stages of examinations (examinations I, II, III) and components of body mass according to the diets applied.

	Sum of Squares	df	Mean Square	F	*p*
**BMI**					
Between groups	0.449	2	0.224	0.010	0.990
Inside groups	6631.363	297	22.328		
Total	6631.812	299			
**FAT [%]**					
Between groups	23.168	2	11.584	0.216	0.806
Inside groups	15961.233	297	53.742		
Total	15984.402	299			
**VFAT [level]**					
Between groups	1.167	2	0.583	0.028	0.973
Inside groups	6232.630	297	20.985		
Total	6233.797	299			
**TBW [%]**					
Between groups	8.588	2	4.294	0.142	0.867
Inside groups	8953.700	297	30.147		
Total	8962.288	299			
**PMM [%]**					
Between groups	7.268	1	7.268	0.149	0.700
Inside groups	9649.901	198	48.737		
Total	9657.169	199			

BMI: Body mass index; FAT: Fat mass; VFAT: Visceral fat; TBW: Body water; PMM: Muscle mass.

**Table 4:** Type of diet and functional fitness after a four-month observation of stroke patients.

	Barthel INDEX		Brunnström Scale		Ashworth Scale		
Diet	General Functional Fitness	Functional Fitness of the Hand	Functional Fitness of the Upper Limb	Functional Fitness of the Lower Limb	Hand Muscle Tone	Hand Muscle Tone	Muscle Tone of the Lower Limb
**Basic diet (D1)**							
Mean	17.86	0.50	0.07	0.14	0.43	0.36	0.14
SD	12.82	0.52	0.27	0.36	0.51	0.63	0.36
Me	17.50	0.50	0.00	0.00	0.00	0.00	0.00
Min	0.00	0.00	0.00	0.00	0.00	0.00	0.00
Max	40.00	1.00	1.00	1.00	1.00	2.00	1.00
**Easily digestible diet (D2)**							
Mean	16.18	0.33	0.25	0.29	0.22	0.18	0.06
SD	10.52	0.48	0.44	0.54	0.54	0.56	0.37
Me	15.00	0.00	0.00	0.00	0.00	0.00	0.00
Min	0.00	0.00	0.00	0.00	−2.00	−1.00	−1.00
Max	45.00	1.00	1.00	2.00	1.00	2.00	1.00
**Diet with restriction of easily digestible carbohydrates (D3)**							
Mean	13.61	0.39	0.39	0.61	0.06	−0.06	−0.06
SD	12.46	0.50	0.61	0.70	0.42	0.42	0.42
Me	12.50	0.00	0.00	0.50	0.00	0.00	0.00
Min	0.00	0.00	0.00	0.00	−1.00	−1.00	−1.00
Max	45.00	1.00	2.00	2.00	1.00	1.00	1.00
**Easily digestible low-protein diet (D4)**							
Mean	12.06	0.41	0.59	0.35	0.65	0.53	0.24
SD	13.93	0.51	0.51	0.49	0.79	0.62	0.44
Me	10.00	0.00	1.00	0.00	0.00	0.00	0.00
Min	0.00	0.00	0.00	0.00	0.00	0.00	0.00
Max	45.00	1.00	1.00	1.00	2.00	2.00	1.00
**Total**							
Mean	15.25	0.38	0.31	0.34	0.29	0.22	0.08
SD	11.79	0.49	0.49	0.55	0.59	0.58	0.39
Me	15.00	0.00	0.00	0.00	0.00	0.00	0.00
Min	0.00	0.00	0.00	0.00	−2.00	−1.00	−1.00
Max	45.00	1.00	2.00	2.00	2.00	2.00	1.00
*p*	0.2538	0.7074	0.0145 *	0.1069	0.0292 *	0.0099 *	0.1590

* Indicate significant values (p < 0.05); SD: Standard deviation; Me: Median; Min: Minimum value; Max: Maximum value.

## Abbreviations

BMI	body mass index
FAT	fat mass
VFAT	visceral fat
PMM	muscle mass
TBW	body water
FFM	fat-free mass
BIA	bioelectrical impedance analysis
